# Dynamic multistage nanozyme hydrogel reprograms diabetic wound microenvironment: synergistic oxidative stress alleviation and mitochondrial restoration

**DOI:** 10.1016/j.mtbio.2025.101780

**Published:** 2025-04-17

**Authors:** Jingyu Yan, Yifan Zhao, Chenying Cui, Lihong Zhou, Yurong Xu, Ziyang Bai, Kaifang Zhang, Jiahui Tong, Yingyu Liu, Lingxiang Sun, Meijun Du, Yanling Mi, Xing Wang, Xiuping Wu, Bing Li

**Affiliations:** Shanxi Medical University School and Hospital of Stomatology and Shanxi Provincial Engineering Research Center for Oral Biomaterials, Taiyuan, 030001, Shanxi, China

**Keywords:** Nanozymes, ROS scavenging, Immunoregulation, Mitochondrial, Wound healing

## Abstract

Chronic diabetic wounds remain a significant clinical challenge due to persistent bacterial infections, oxidative stress, impaired angiogenesis, and mitochondrial dysfunction. Traditional therapies often fail to address these interrelated pathological factors, highlighting the urgent need for innovative solutions. Here, we present a Mn-ZIF@GOx/BC (MZGB) hydrogel system, where Mn-ZIF@GOx (MZG) nanozymes are successfully integrated into a bacterial cellulose (BC) hydrogel via hydrogen bonding and electrostatic interactions. The MZGB hydrogel lowers wound pH by oxidizing excess glucose into gluconic acid. It exhibits strong ROS scavenging capabilities through its superoxide dismutase and catalase-like activities, while simultaneously providing oxygen. By restoring redox homeostasis, it protects mitochondrial function and enhances cellular energy metabolism. By reprogramming macrophages, MZGB creates a favorable immune microenvironment, significantly promoting angiogenesis through paracrine mechanisms. This facilitates cell-to-cell communication, forming a positive feedback loop. Moreover, MZGB demonstrates ROS-independent antibacterial properties. BC hydrogel ensures adhesion and moisture regulation, forming a protective barrier and maintaining an optimal wound environment. This multifunctional hydrogel represents a promising nanotherapeutic approach for efficiently treating diabetic wounds by precisely regulating the wound microenvironment.

## Highlights

• MZGB synergistically regulates the wound microenvironment.
• MZGB lowers pH, scavenges ROS, and restores mitochondrial function for healing.
• Reprograms macrophages to boost angiogenesis via immune modulation.
• Exhibits ROS-independent antibacterial activity for broad protection.
• BC hydrogel maintains moisture and adhesion, forming an optimal wound barrier.

## Graphical abstract

**Figure:**
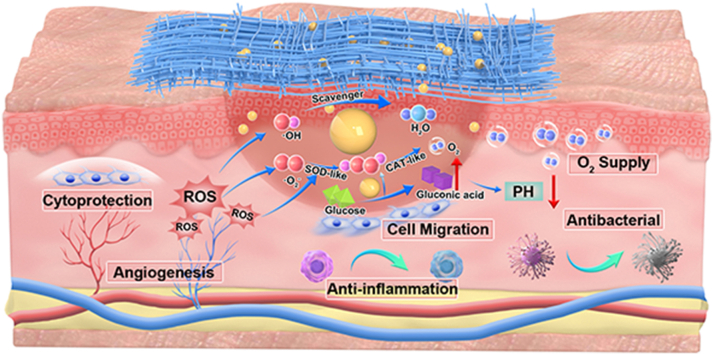

## 1 Introduction

Diabetes, a chronic metabolic disorder marked by persistent hyperglycemia, presents a significant global health challenge with its escalating prevalence and associated complications [1]. Among these myriad complications, diabetic wounds pose a substantial clinical burden due to the risks of uncontrolled bacterial infection, persistent inflammation, impaired angiogenesis, disrupted oxygen delivery, dysregulated reactive oxygen species (ROS) levels, and the heightened risk of mitochondrial dysfunction [2].

In chronic diabetic wounds, persistent bacterial infections, hyperglycemia, and ROS accumulation triggered by mitochondrial dysfunction are key factors contributing to delayed wound healing [3]. These factors exacerbate the wound-healing process by inducing sustained inflammation, vascular endothelial dysfunction, and tissue necrosis [4]. In a hyperglycemic environment, mitochondrial metabolic dysregulation leads to excessive leakage of mitochondria-derived ROS (mtROS), further intensifying oxidative stress [5]. This oxidative stress not only disrupts macrophage polarization but also drives prolonged inflammatory responses, ultimately resulting in microvascular damage and tissue functional decline [6]. Additionally, chronic bacterial infections represent another critical challenge, significantly impairing wound healing by perpetuating inflammation and promoting tissue necrosis [7,8].

Conventional therapeutic strategies, such as debridement and antibiotic therapy, can provide some degree of antimicrobial action and alleviate lesions in the ulcerated area. However, they often fail to address the underlying pathophysiological issues related to ROS dysregulation [9]. While exogenous antioxidants have shown promise in mitigating oxidative stress [[10], [11], [12]], their limited stability, bioavailability, and specificity pose significant challenges for clinical translation. Therefore, innovative therapeutic strategies are urgently needed to effectively regulate ROS levels in the wound microenvironment, restore mitochondrial function, promote angiogenesis, and simultaneously exhibit antibacterial properties, thereby facilitating comprehensive healing of diabetic wounds.

Recent advancements in nanozyme research have highlighted their enzyme-mimicking catalytic properties, presenting a promising alternative to traditional antioxidants [13]. Nanozymes can replicate the functions of natural antioxidant enzymes, such as superoxide dismutase (SOD) and catalase (CAT), to eliminate ROS while offering superior stability, cost-efficiency, and biocompatibility [14]. Among these, manganese-based nanozymes (Mn-nanozymes) stand out for their ability to mimic multiple antioxidant enzymes, maintain redox balance, and mitigate excessive inflammation [15].

However, the spatial variability of ROS levels across different cell types poses a challenge. While physiological ROS levels are essential for endothelial and fibroblast function, macrophages and neutrophils rely on elevated ROS to eliminate pathogens [16,17]. The spatial variation in ROS levels suggests that reducing ROS to physiological ranges will inevitably compromise their ROS-dependent antimicrobial functions [18]. Thus, nanozymes with ROS scavenging abilities and ROS-independent antibacterial properties offer a promising strategy for diabetic wound treatment.

Metal-organic frameworks (MOFs) are coordination networks with tunable pores and channels, offering enzyme-like hydrophobic environments [19,20]. Their catalytic properties can be tailored by selecting specific metal ions and ligands, making them versatile for developing ROS-scavenging nanozymes [21]. Zeolitic imidazolate frameworks (ZIFs), a subclass of MOFs, feature zeolite-like topology. Among them, ZIF-8 exhibits antibacterial properties by releasing organic linkers and zinc ions [22].

Research shows that diabetic wounds typically exhibit an alkaline pH (7.0–8.9) [23], which hinders the natural healing process. Regulating wound pH is crucial for effective healing [24,25]. Glucose oxidase (GOx) catalyzes glucose oxidation to produce hydrogen peroxide (H_2_O_2_) and gluconic acid, lowering pH and generating H_2_O_2_, which induces various biological effects [26]. This dual action creates a microenvironment favorable for healing.

Bacterial cellulose (BC), a natural biopolymer synthesized by certain bacteria (such as *Acetobacter xylinum*), possesses a unique three-dimensional nanofibrous network, high mechanical strength, excellent biocompatibility, and remarkable water retention capacity, making it an ideal carrier for nanozymes [27]. BC not only effectively stabilizes nanozymes through hydrogen bonding and electrostatic interactions, preventing rapid release or aggregation, but also maintains the activity and functionality of nanozymes over prolonged periods in complex biological environments due to its outstanding mechanical properties and structural integrity [28]. The porous architecture of BC plays a crucial role in enhancing oxygen permeability and promoting cell migration, while its high specific surface area facilitates uniform nanozyme distribution and sustained release, thereby synergistically enhancing local catalytic activity and antibacterial effects [29]. Furthermore, the continuous network structure of BC firmly adheres to the wound surface, forming a protective barrier that effectively absorbs exudates and maintains a moist environment, thereby promoting tissue regeneration and healing [30]. While other carrier materials, such as synthetic hydrogels and natural polysaccharides, may offer some similar benefits, they often fall short of fully replicating the unique advantages of BC. Synthetic hydrogels, though customizable in properties, may lack the inherent biocompatibility and biodegradability of BC and could introduce potential biotoxicity concerns [28]. Natural polysaccharides like alginate and chitosan, while biocompatible, typically exhibit inferior mechanical strength and structural stability compared to BC. The distinctive combination of BC's mechanical robustness, structural integrity, and nanofibrous architecture creates an optimal environment for nanozyme functionality and wound healing, which is difficult to achieve with alternative carriers. However, its efficacy in promoting diabetic wound healing is limited, lacking the ability to regulate environmental factors for targeted treatment. To address this, we aim to develop a BC hydrogel incorporating MOF nanozymes, combining BC's benefits with nanozymes' properties to precisely regulate the wound environment and enhance diabetic wound treatment.

**Scheme 1:**
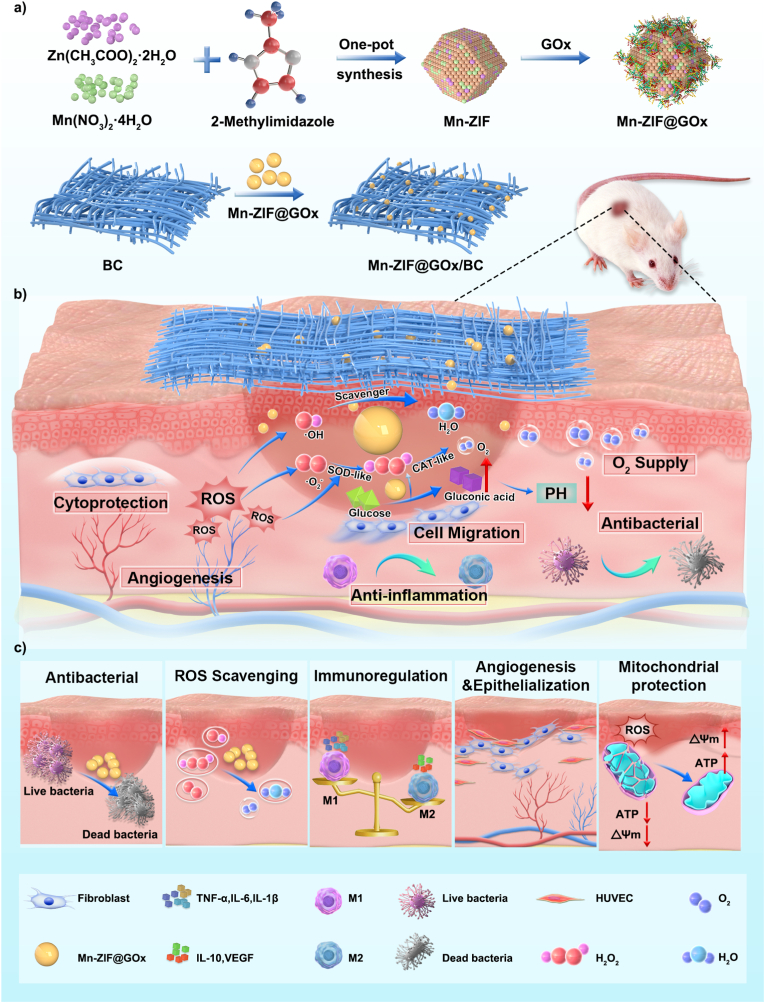
The preparation of MZGB hydrogel and the multifunctional roles of MZGB hydrogel in the diabetic wound healing process.

This study introduces the Mn-ZIF@GOx/BC (MZGB) hydrogel system, a multifunctional bioactive hydrogel created by incorporating Mn-ZIF@GOx (MZG) nanozymes into a BC hydrogel (Scheme 1). The MZGB hydrogel offers the following advantages: (1) Reducing pH at the local wound site: Utilizing abundant glucose *in vivo*, MZG can consume excess glucose and oxidize it to gluconic acid. (2) Multiple ROS scavenging capabilities: MZG effectively eliminates hydroxyl radicals (•OH), superoxide anions (O_2_^•−^), and H_2_O_2_ through a cascade process simulating SOD and CAT, while also providing oxygen. (3) Mitochondrial protection: The hydrogel restores ATP production, reestablishes redox balance, and mitigates oxidative stress damage. (4) Immunomodulation: MZG can stimulate macrophage polarization towards the M2 phenotype, facilitating an anti-inflammatory microenvironment. (5) Promoting angiogenesis: Through paracrine mechanisms, significant promotion of endothelial cell and fibroblast proliferation, migration, and *in vitro* angiogenesis of endothelial cells is observed. (6) ROS-independent antimicrobial performance: MZG undergoes dissociation via coordination breakdown, with Zn^2+^ exhibiting potent antimicrobial properties. BC hydrogel exhibits excellent biocompatibility, effectively adhering to wound surfaces to form a protective barrier against bacterial infections. With its ROS-scavenging capabilities and potential for modulating the diabetic wound immune microenvironment, the MZGB hydrogel offers a promising strategy for the treatment of diabetic wounds.

## 2 Materials and methods

### 2.1 Synthesis of MZG

To synthesize Mn-ZIF, Zn(CH_3_COO)_2_ •2H_2_O (0.2 M) and Mn(NO_3_)_2_•4H_2_O (0.04 M) were dissolved in methanol and mixed with 2-methylimidazole (1 M). After 5 h of stirring, the mixture was centrifuged, and the precipitate was washed and vacuum-dried. For MZG formation, Mn-ZIF was dissolved in water with GOx (50 mg) and stirred at 30 °C for 24 h. The precipitate was then centrifuged, washed, and vacuum-dried to obtain MZG.

### 2.2 Preparation of the BC and MZGB

*Acetobacter xylinum* NUST4 was initially cultured under constant temperature shaking (30 °C, 200 rpm) for 24 h. The culture was transferred to a fermentation solution and incubated further to form BC membranes, which were harvested every 12–24 h. BC was purified by washing with deionized water, boiling in 0.1 M NaOH, and rinsing until neutral. A hydrogel composed of BC and MZG (abbreviated as MZGB) was prepared. For MZGB1, BC (1 g) was dispersed in 20 mL ultrapure water, followed by the addition of 5 mL MZG (20 mg/mL). The mixture was shaken at 160 rpm and 30 °C for 24 h, then rinsed with deionized water. MZGB2 and MZGB3 were prepared similarly, using MZG concentrations of 40 mg/mL and 80 mg/mL, respectively, with all other steps identical to those for MZGB1.

### 2.3 Characterization

The synthesized nanomaterials and hydrogels (MZG, BC, and MZGB) were comprehensively characterized. Morphological features were analyzed via scanning electron microscopy (SEM), and particle size distribution was assessed using dynamic light scattering (DLS). Transmission electron microscopy (TEM) revealed the nanoscale architecture, while energy-dispersive X-ray spectroscopy (EDS) confirmed the presence and homogeneous distribution of metal elements. Elemental composition and chemical states were examined through X-ray photoelectron spectroscopy (XPS), while Fourier-transform infrared spectroscopy (FT-IR) identified functional groups. Thermal stability was evaluated using thermogravimetric analysis (TGA), and structural properties were investigated via X-ray diffraction (XRD). Additionally, zeta potential measurements determined surface charge, and contact angle goniometry assessed surface wettability. Adhesion testing involved applying the MZGB hydrogel (3 cm × 1.5 cm) to a subject's finger joint, rotating it at varying angles (0°, 90°, 120°, and 180°), and capturing photographic documentation. The study was approved by the Shanxi Medical University Medical Ethics Committee (Approval No. 2024SLL042), with informed consent obtained from the subject.

### 2.4 Ion release behavior

Each sample was immersed in simulated body fluid (SBF) with pH values of 6.0 and 7.4, respectively, and placed in a constant-temperature water bath shaker at 37 °C. At predetermined time intervals, the released ions were collected by removing the liquid from the centrifuge tubes, followed by replenishment with fresh SBF. The concentrations of Zn^2+^ and Mn^2+^ released from the samples at each time point were analyzed using inductively coupled plasma optical emission spectrometry (ICP-OES).

### 2.5 ROS scavenging ability

Based on the fact that GOx can catalyze glucose to generate gluconic acid, the cascade catalytic activity was evaluated by monitoring glucose consumption and pH reduction in the reaction system. To investigate the catalytic performance of MZG under simulated diabetic wound microenvironment conditions, a glucose solution (1 mg/mL) and MZG (50 μg/mL) were added to PBS buffer (pH 7.8). At predetermined time intervals, 50 μL of the reaction solution was collected and immediately mixed with 100 μL of 1 % DNS solution. The mixture was then heated in a boiling water bath at 100 °C for 5 min, followed by rapid cooling in ice water. After cooling, the absorbance at 540 nm was measured using a microplate reader. The residual glucose concentration was calculated according to the glucose standard curve. Simultaneously, pH variation in the reaction system was monitored under the same experimental conditions by directly measuring the pH value of the reaction solution at different time points using a digital pH meter.

The SOD-like activity was assessed using the NADH-mPMS-NBT assay, while CAT-like activity was evaluated by measuring the oxygen content in the buffer solution. Furthermore, electron spin resonance (ESR) spectroscopy was employed to verify the •OH scavenging ability of MZG.

### 2.6 Free radical scavenging assays

Firstly, the ABTS assay was employed to assess the antioxidant potential of MZG. ABTS reacts with oxidants to produce green-colored ABTS^•+^. In the presence of antioxidants, the formation of ABTS^•+^ is inhibited. The absorbance of ABTS^•+^ can be measured at 734 nm or 405 nm to determine and calculate the total antioxidant capacity of the samples. Additionally, the DPPH free radical scavenging assay was conducted to further validate the antioxidant activity. DPPH in ethanol solution appears purple and exhibits strong absorption at 515 nm. In the presence of antioxidants, DPPH radicals are scavenged, resulting in a lighter color of the solution and a decrease in absorbance at 515 nm. The change in absorbance is proportional to the extent of DPPH radical scavenging and is measured to calculate the DPPH radical scavenging percentage of the samples.

### 2.7 Antibacterial performance

*Escherichia coli* (*E. coli*), *Staphylococcus aureus* (*S. aureus*), and methicillin-resistant *Staphylococcus aureus* (MRSA) were selected as target strains to evaluate the antibacterial properties of different groups. The prepared bacterial suspensions were diluted to a concentration of 1 × 10^8^ CFU/mL, followed by the addition of 100 mg of the sample to 1 mL of the bacterial suspension. After incubation for 24 h, 30 μL of the bacterial solution was evenly spread onto agar plates and incubated overnight at 37 °C. Bacterial growth was monitored by counting colony-forming units and photographing the agar plates.

Following treatment with the different groups, the bacterial suspensions were collected and washed three times with PBS to remove residual substances. Subsequently, the bacteria were fixed with 2.5 % glutaraldehyde at 4 °C overnight. The fixed samples were then subjected to a graded ethanol dehydration series (25 %, 50 %, 75 %, and 100 %), followed by drying and coating with a thin layer of gold for SEM analysis to observe bacterial morphology.

For the antibacterial zone of inhibition assay, bacterial suspensions at a concentration of 1 × 10^7^ CFU/mL were evenly inoculated onto agar plates using sterile cotton swabs. Sterilized BC and MZGB nanocomposite membranes were cut into 6 mm diameter discs and placed on the agar plates containing the designated bacterial strains. The plates were incubated at 37 °C for 24 h, and the inhibition zones were observed and photographed to evaluate the antibacterial effect.

Bacterial viability was assessed using a live/dead bacterial staining kit (SYTO9-PI). After treatment with different hydrogels for 24 h, the bacterial suspensions were processed and stained according to the manufacturer's instructions. The stained samples were then observed using CLSM to evaluate bacterial viability.

To assess the inhibitory effect of the materials on biofilm formation, the crystal violet staining method was employed. The different materials were incubated with bacterial suspensions (1 × 10^7^ CFU/mL) for 24 h. After incubation, the supernatant was discarded, and the wells were washed three times with PBS to remove planktonic bacteria. The biofilms were then stained with 0.1 % crystal violet solution for 15 min, followed by thorough washing to remove excess dye and subsequent drying at 37 °C. The stained biofilms were solubilized with 200 μL of absolute ethanol, and the absorbance was measured at 570 nm using a microplate reader to calculate the biofilm disruption rate.

### 2.8 Intracellular ROS scavenging capacity and mitochondrial regulatory function assessment

Intracellular ROS levels were evaluated using the ROS indicator 2′,7′-dichlorodihydrofluorescein diacetate (DCFH-DA). Human umbilical vein endothelial cells (HUVECs), L929 mouse fibroblast cell line (L929 cells), and RAW264.7 murine macrophage cell line (RAW264.7 cells) were seeded in 24-well plates at a density of 2 × 10^4^ cells per well. After treatment with H_2_O_2_ and hydrogels for 24 h, the cells were washed with PBS. Subsequently, each well was incubated with the DCFH-DA probe for 30 min, followed by washing three times with a basal medium. The fluorescence intensity of ROS was observed using confocal microscopy and analyzed by flow cytometry. The mtROS levels were measured using the same procedure.

The mitochondrial membrane potential (MMP) was assessed using the JC-1 assay kit to evaluate the effects of the hydrogel on mitochondrial function. Intracellular ATP levels were quantified using an ATP assay kit.

### 2.9 Quantitative real-time PCR (qRT-PCR)

The mRNA expression levels of IL-1*β*, IL-6, TNF-*α*, IL-10, Arg-1, and VEGF were measured using a qRT-PCR instrument. All mRNA expression levels were normalized to the reference gene GAPDH. The relative mRNA expression levels of the target genes were calculated relative to the control samples using the 2^−ΔΔCt^ method. The sequences of the forward and reverse primers are provided in Table S1.

### 2.10 Preparation of macrophage-conditioned medium (CM)

RAW 264.7 cells were pre-treated with LPS (100 ng/mL) for 8 h in 6-well plates. The cells were then treated with BC and MZGB hydrogels for 24 h, followed by replacing the medium with fresh DMEM containing 10 % FBS and 1 % penicillin-streptomycin. After an additional 24 h of incubation, the culture medium was collected and centrifuged at 12,000 rpm for 15 min to remove cell debris and particulates. The supernatant was then filtered through a 0.22 μm filter to obtain the macrophage CM.

### 2.11 Cell scratch, migration, and tube formation assays

Cell scratch assays were conducted on HUVECs to simulate wound healing, with images taken at 0 and 24 h to assess the closure of the scratch. The migratory capacity of HUVECs and L929 cells was assessed via a Transwell migration assay, with cells stained and photographed after 24 h. *In vitro* tube formation was evaluated by seeding HUVECs pre-treated with conditioned media on Matrigel-coated plates, and tube formation was observed and analyzed after 6 h.

### 2.12 Evaluation of *in vivo* diabetic wound healing

Male SD rats were obtained from the experimental animal center of Shanxi Medical University. All animal experiments were approved by the Animal Ethics Committee of Shanxi Medical University (Ethics Approval No.: KQDW-2024-004). All animal procedures were conducted following the U.K. Animals (Scientific Procedures) Act of 1986 and its relevant guidelines, as well as the ARRIVE guidelines. Firstly, the diabetic wound model was established. To evaluate MZGB hydrogel efficacy, diabetic rats were treated with normal saline, BC hydrogel, or MZGB hydrogel, and wound healing was monitored through digital imaging and analyzed using Image J. Wound tissues were harvested for histological examination, including H&E and Masson's trichrome staining on days 7 and 14. Additionally, ROS levels, macrophage polarization, inflammation, and angiogenesis were evaluated using targeted staining methods. To further evaluate the *in vivo* antibacterial efficacy of the MZGB hydrogel, an MRSA bacterial suspension (10 μL, 1 × 10^8^ CFU/mL) was directly applied to the wound area. MRSA colony counting was performed on the wound tissues on day 7 post-treatment.

### 2.13 Statistical analysis

All statistical analyses were performed using GraphPad Prism. Results are expressed as the mean ± standard deviation. The significance of differences was denoted as follows: ns represents *p* > 0.05, ∗ represents *p* < 0.05, ∗∗ represents *p* < 0.01, ∗∗∗ represents *p* < 0.001, and ∗∗∗∗ represents *p* < 0.0001.

## 3 Results and discussion

### 3.1 Synthesis and characterization of MZG

**Fig. 1:**
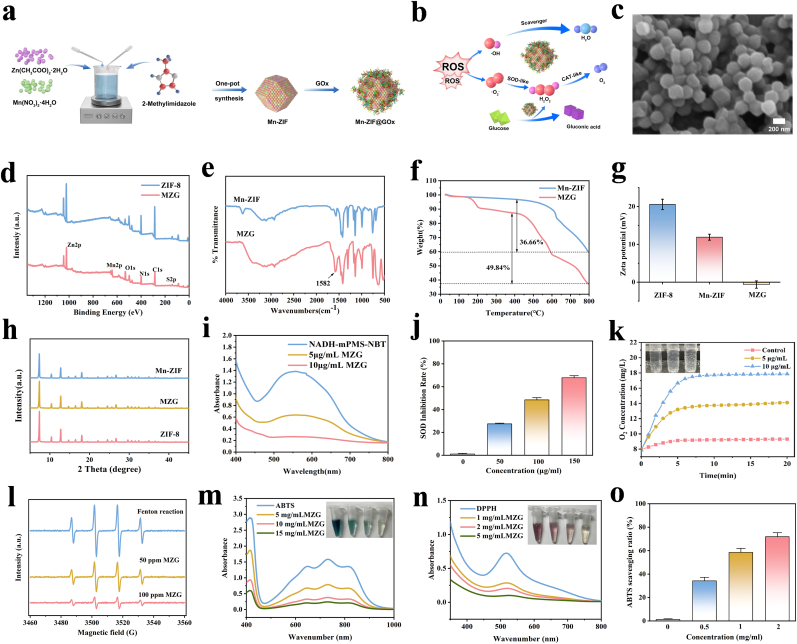
Physicochemical characteristics and ROS scavenging capacity of MZG. (a) The synthesis of MZG. (b) The enzyme-like activities of MZG. (c) SEM image of MZG. (d) XPS spectra. (e) FT-IR spectra. (f) TGA curve. (g) Zeta potential. (h) XRD patterns. (i) NADH-mPMS-NBT assay to detect the SOD-like activity of MZG. (j) Comparison of the SOD-like activity of MZG. (k) Variation in the amount of O_2_ generated by the catalysis of H_2_O_2_ by MZG. (l) DMPO/•OH ESR spectra of MZG. (m) Absorbance curve of the ABTS assay. (n) Absorbance curve of the DPPH assay. (o) ABTS scavenging rate of MZG.

Mn-ZIF was synthesized using a solvothermal approach. The Mn-ZIF produced displays a rhombic dodecahedral shape (Fig. S1a), resembling the morphology of ZIF-8 synthesized in the absence of Mn ions (Fig. S1b). In the formation of Mn-ZIF, Mn ions interact with 2-methylimidazole, integrating into the nanoscale ZIF structure and leading to a stable and uniform distribution of Mn ions. Subsequently, GOx is embedded into the uniformly sized Mn-ZIF crystals through a simple co-precipitation method, successfully yielding MZG (Fig. 1a,c). Water was used as the solvent during the preparation process, which effectively preserved the activity of GOx. The size of MZG was further analyzed using DLS (Fig. S2), with results closely matching the observations from SEM. This consistency indicates that the nanocomposite has good dispersibility in aqueous solution, suggesting its potential for biomedical applications.

The EDS mapping shows the distribution of Mn, Zn, and S elements within the material (Fig. S3). Since S is a characteristic element of proteins, the EDS results demonstrate the successful attachment of GOx onto the Mn-ZIF. Subsequently, XPS analysis of MZG was performed, revealing characteristic peaks for Zn 2p, Mn 2p, N 1s, O 1s, C 1s, and S 2p (Fig. 1d). The presence of Zn 2p was indicated by two prominent peaks at 1021.8 eV and 1044.6 eV (Fig. S4a). Additionally, the Mn 2p spectrum exhibited two peaks at 653.8 eV and 642.0 eV (Fig. S4b), corresponding to Mn 2p_1/2_ and Mn 2p_3/2_, respectively. In the Mn 2p_3/2_ XPS spectrum, two peaks were observed at binding energies of 640.5 eV and 642.1 eV, which correspond to Mn^2+^ and Mn^4+^, respectively. This indicates that Mn exists as a mixture of Mn^2+^ and Mn^4+^ within the Mn-ZIF framework. Additionally, two binding energies at 161 eV and 162.2 eV were observed (Fig. S4c), attributed to the signals of S 2p. Since sulfur is a characteristic element of proteins, these results indicate that GOx has been successfully embedded into Mn-ZIF.

Comparing the FT-IR spectra of MZG with Mn-ZIF (Fig. 1e), the peak at 1582 cm^−1^ further confirms the incorporation of GOx. To determine the thermal stability of MZG, TGA curves were measured (Fig. 1f). MZG displayed a gradual weight loss of approximately 8.9 % at around 200 °C, reflecting the evaporation of water and other small organic molecules from the MOF cavities. Upon further heating, Mn-ZIF underwent a second stage of decomposition, resulting in a weight loss of about 36.66 %. In the same temperature range, MZG showed a weight loss of approximately 49.84 % due to the decomposition of both Mn-ZIF and GOx. Upon loading negatively charged GOx onto Mn-ZIF, a shift towards negative charges was observed (Fig. 1g). These results further confirm the successful synthesis of MZG. The XRD pattern of MZG exhibits characteristic diffraction peaks consistent with those of both ZIF-8 and Mn-ZIF (Fig. 1h), indicating that the incorporation of Mn and GOx does not disrupt the crystal structure of the ZIF-8 framework. Notably, the characteristic peaks corresponding to the ZIF-8 are observed in MZG, demonstrating that the specific crystal structure of the material is well preserved after functionalization. This structural integrity is crucial for maintaining the intrinsic bioactivity of the encapsulated protein.

### 3.2 Evaluation of ROS scavenging capacity

Persistent inflammation and high glucose conditions in diabetic wounds generate an overabundance of ROS, resulting in oxidative damage [31]. This study aims to eliminate excess ROS and modulate the diabetic wound microenvironment (Fig. 1b). The primary ROS include H_2_O_2_, O_2_^•−^, and •OH. The SOD-mimetic activity of MZG was evaluated utilizing the NADH-mPMS-NBT system (Fig. 1i). In this system, the reaction between NADH and mPMS generates a significant amount of O_2_^•−^, which oxidizes NBT, leading to a characteristic UV absorption peak at 560 nm. Adding MZG to the NADH-mPMS-NBT system significantly decreased the absorption peak at 560 nm, indicating MZG's ability to scavenge O_2_^•−^. Furthermore, increasing concentrations of MZG enhanced the SOD inhibition rate from 27.47 % to 67.84 % (Fig. 1j), demonstrating a dose-dependent clearance of O_2_^•−^ by MZG. Subsequently, MZG was added to a buffered solution containing H_2_O_2_, where it catalyzed the production of O_2_. Monitoring changes in dissolved oxygen levels with a dissolved oxygen meter revealed a continuous increase in oxygen levels upon the addition of MZG. With increasing concentrations of MZG, the rate of H_2_O_2_ decomposition accelerated, significantly elevating dissolved oxygen levels (Fig. 1k). The ability of MZG to scavenge •OH was further validated using ESR spectroscopy. The ESR spectrum (Fig. 1l) exhibited four distinctive peaks with an intensity ratio of 1:2:2:1, characteristic of spin adducts formed by DMPO reacting with •OH. The signal intensity correlates with the concentration of •OH. Upon addition of MZG to the free radical system, the DMPO/•OH signal noticeably attenuated, indicating the effective scavenging capability of MZG towards •OH.

The antioxidant capacity of MZG was further evaluated using the ABTS radical scavenging assay. As shown in Fig. 1m, ABTS can be oxidized to ABTS^•+^, resulting in a blue-green solution with characteristic absorption peaks at 405 nm and 734 nm. Upon the addition of MZG, the ABTS^•+^ is reduced, changing the solution color from blue-green to colorless and resulting in a decrease in absorbance, demonstrating its strong antioxidant capability. Additionally, the clearance of ABTS^•+^ by MZG is concentration-dependent. As the concentration increased, the clearance rates of ABTS^•+^ by MZG improved from 34.22 % to 71.95 % (Fig. 1o), demonstrating a dose-dependent inhibition of ABTS radical formation by MZG. Similarly, the ethanol solution of DPPH appears purple with a characteristic absorption peak at 517 nm. Upon reaction with MZG, a decrease in the absorption peak is observed, indicating significant decolorization of the DPPH solution (Fig. 1n). The DPPH scavenging capacity of MZG also shows concentration dependence. As the concentration of MZG rises, the DPPH scavenging rates by MZG increase from 38.03 % to 82.03 % (Fig. S5).

Additionally, cascade catalytic reaction experiments were performed using MZG. Taking advantage of the ability of GOx to catalyze the conversion of glucose into gluconic acid and H_2_O_2_, we first monitored glucose consumption and pH variation during the reaction process (Fig. S6). The experimental results demonstrated that, with increasing reaction time, glucose was gradually depleted, accompanied by a significant decrease in pH, confirming the effective catalysis of glucose to gluconic acid by GOx. Subsequently, we systematically compared the differences in ROS scavenging capacities between MZG and the glucose + MZG composite system, with a particular focus on CAT activity, SOD activity, and •OH scavenging ability. In the glucose + MZG system, CAT activity was significantly enhanced, which could be attributed to two main factors: the activation of nanozyme catalysis under acidic conditions and the increased substrate concentration resulting from H_2_O_2_ generation via GOx-mediated glucose oxidation (Fig. S7b). In parallel, the •OH scavenging ability was markedly improved under acidic conditions (Fig. S7c). However, in contrast, SOD activity exhibited a slight decline under acidic conditions, possibly due to the suppression of the superoxide dismutation reaction in an acidic microenvironment (Fig. S7a). These findings indicate that regulating local pH and H_2_O_2_ generation through GOx-catalyzed reactions can effectively augment the ROS scavenging performance of nanozymes within diabetic wound environments, thereby enhancing their antioxidant efficacy under oxidative stress conditions.

### 3.3 Synthesis and characterization of MZGB hydrogels

**Fig. 2:**
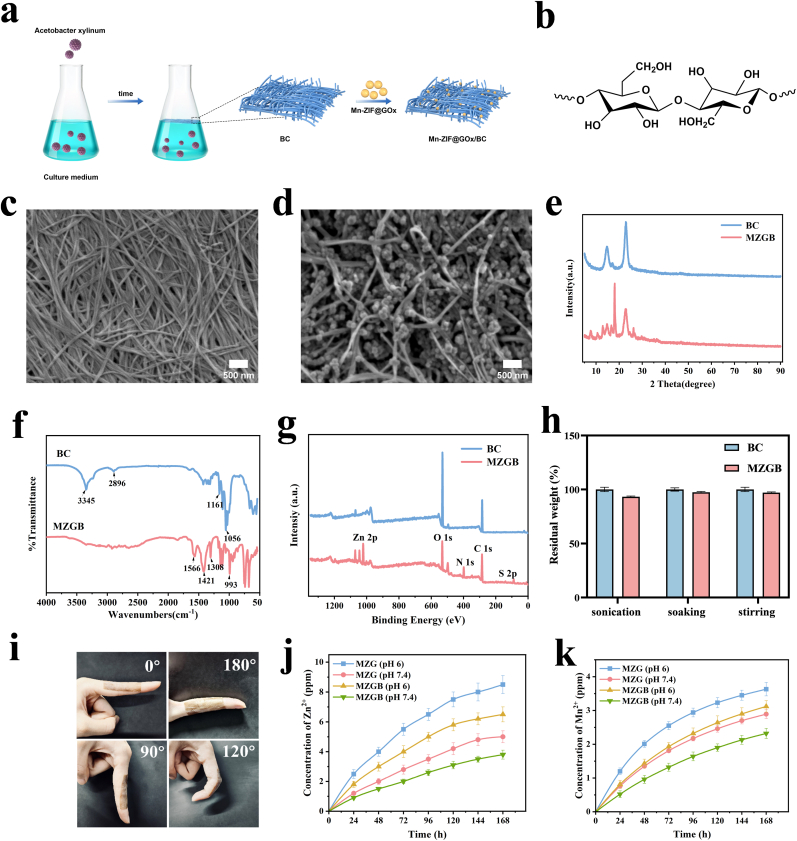
Physicochemical structure characterization of MZGB hydrogels. (a) The synthesis process for MZGB hydrogels. (b) The structure of BC. (c, d) SEM image of BC and MZGB. (e, f, g) XRD, FT-IR, and XPS spectra of BC and MZGB. (h) Fastness evaluation of embedded MZG. (i) Digital camera photographs of MZGB hydrogel adhesion. (j,k) Cumulative release of Zn^2+^ and Mn^2+^ from MZG and MZGB.

In this study, BC was synthesized by inoculating *Acetobacter xylinum* NUST4 into a fermentation medium under constant temperature shaking, followed by harvesting, washing, boiling in NaOH to remove impurities, and rinsing with deionized water until neutral. Subsequently, MZG nanozymes were integrated with the BC hydrogel to form a multifunctional composite hydrogel (Fig. 2a). Fig. 2b illustrates the structure of BC, where each glucose unit contains multiple hydroxyl groups capable of interacting with MZG. MZG is dispersed and embedded within the fibrous network of BC through electrostatic interactions, hydrogen bonding, and physical adsorption. SEM images depict the well-defined three-dimensional network structure of pure BC (Fig. 2c), which facilitates the entry of nanoparticles into its cavities. Upon functionalization of BC with MZG nanoparticles, the nanoparticles are observed to be deposited on or around the nanofibers (Fig. 2d). XRD analysis was conducted to investigate the crystalline properties of BC and MZGB (Fig. 2e). Diffraction peaks in the XRD pattern of BC correspond to crystal planes (110) and (200), which are also observed in the MZGB composite, indicating that the crystalline properties of BC and MZG remain unchanged after composite formation. As shown in the FT-IR spectrum in Fig. 2f, the BC spectrum exhibits intense peaks at 3345 cm^−1^, 2896 cm^−1^, 1056 cm^−1^, and 1161 cm^−1^, which correspond to O-H, C-H, C-O, and C-O-C stretching vibrations, respectively. The FT-IR spectrum of MZGB shows characteristic peaks at 1566 cm^−1^, 1421 cm^−1^, 993 cm^−1^, and 1308 cm^−1^, corresponding to C=N stretching, C-N stretching, and imidazole bending vibrations. The XPS analysis of MZGB (Fig. 2g) further confirms the successful incorporation of MZG into BC. We further evaluated the stability of MZGB in solution, and after various treatments, the leaching rate of MZG in solution was less than 8 % (Fig. 2h), indicating that MZG exhibits high stability within the BC matrix. Furthermore, the hydrophilicity of materials, a crucial factor for cell adhesion, can be evaluated through contact angle measurements, as demonstrated in Fig. S8. Additionally, hydrogels used for treating diabetic skin defects require excellent adhesion properties. As depicted in Fig. 2i, MZGB adheres well to the model finger and can be rotated without detachment, indicating strong adhesive properties.

Given the previous observation that the reaction between glucose and MZG results in a significant decrease in pH over time, we investigated the release profiles of Zn^2+^ and Mn^2+^ from MZG and MZGB under two pH conditions (pH 6 and pH 7.4) over a period of 7-days using ICP-OES. This study aimed to simulate the physiological environment that the materials might encounter during practical applications. As shown in Fig. 2j and k, the release profiles of Zn^2+^ and Mn^2+^ exhibited a distinct pattern characterized by an initial burst release followed by a sustained and gradual release phase. The rapid release observed in the early stage can be attributed to the dissolution of ions loosely bound or physically adsorbed on the material surface. Subsequently, the sustained release phase likely results from the gradual degradation of the material, enabling continuous ion release. Notably, under both pH conditions, the release rates of Zn^2+^ and Mn^2+^ from MZGB were consistently lower than those from MZG. This difference may be attributed to the incorporation of BC, which altered the structural characteristics and diffusion dynamics of the composite material. The interconnected network structure formed by BC potentially increased the tortuosity of the ion diffusion pathway, thereby effectively reducing the ion release rate. Moreover, the pH of the release medium exerted a significant influence on the ion release behavior. At acidic pH (pH 6), both MZG and MZGB exhibited higher release rates of Zn^2+^ and Mn^2+^ compared to neutral conditions (pH 7.4). This pH-responsive release behavior can be ascribed to the protonation of the framework components, which weakens the coordination bonds between metal ions and organic ligands, promoting the dissociation and release of Zn^2+^ and Mn^2+^ ions. Acidic conditions may induce structural changes within the material, accelerating its degradation and enhancing ion release. In summary, the incorporation of BC in MZGB resulted in a decelerated ion release profile compared to MZG, while acidic conditions significantly accelerated the release.

### 3.4 Evaluation of antibacterial activity of MZGB *in vitro*

The above results demonstrate the excellent ROS scavenging capability of MZG. Considering that the regional heterogeneity of ROS indicates that scavenging ROS might compromise ROS-dependent antibacterial properties [32,33], we examined whether MZGB exhibits its antibacterial activity through a ROS-independent mechanism.

**Fig. 3:**
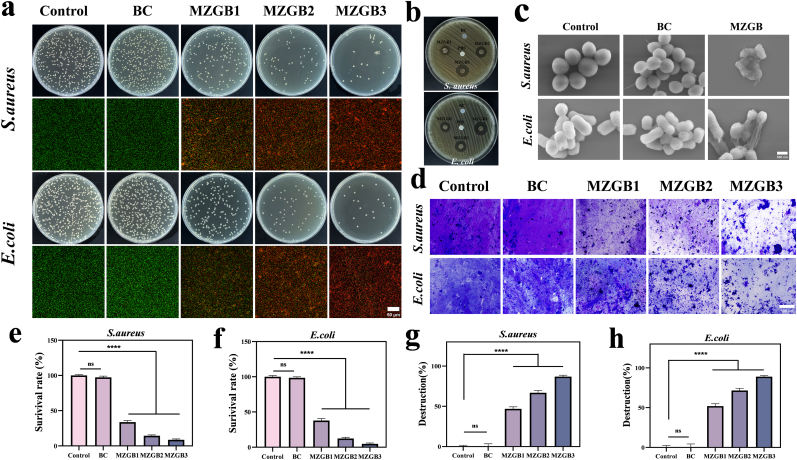
Evaluation of the antibacterial performance of MZGB. (a, e, f) Antibacterial performance of MZGB was assessed by plate colony counting and live/dead bacterial staining, with statistical analysis of the survival rates by plate colony counting. (b) Representative images of inhibition zones for *S. aureus* and *E. coli*. (c) SEM images. (d, g, h) Bacterial biofilms under different treatment conditions and corresponding biofilm disruption rates.

To investigate the antibacterial activity of MZGB, we conducted experiments on three bacterial strains on a cellulose surface: *S. aureus*, *E. coli*, and MRSA. In the *in vitro* bacterial assays, bacterial suspensions were applied to the control group, BC, and MZGB samples at different concentrations. Bacteria were collected from the cellulose using PBS buffer solution (pH 7.4), and their viability was evaluated through plate counting and live/dead staining methods (Fig. 3a, S9). Compared to the control group and BC, the number of *S. aureus*, *E. coli*, and MRSA significantly decreased after MZGB treatment. Following treatment with MZGB1, MZGB2, and MZGB3, the relative survival rates of *S. aureus* were 33.67 %, 14.44 %, and 8.64 %, respectively (Fig. 3e). For *E. coli*, the relative survival rates were 37.75 %, 12.38 %, and 4.86 %, respectively (Fig. 3f). Notably, MRSA also exhibited a substantial reduction in viability after MZGB treatment, with survival rates of 47.29 %, 14.71 %, and 4.15 % for MZGB1, MZGB2, and MZGB3, respectively (Fig. S9). To further assess the antibacterial efficacy of MZGB, we performed an inhibition zone assay against *S. aureus* and *E. coli*. As shown in Fig. 3b, MZGB exhibited significant antibacterial activity, forming clear inhibition zones around the MZGB samples. The diameter of the inhibition zones increased with higher MZG concentrations, confirming the dose-dependent antibacterial effect of MZGB (Fig. S10). Live/dead staining of bacteria (Fig. 3a,S9) revealed that nearly all bacteria in the control and BC groups were stained green. In contrast, *S. aureus*, *E. coli*, and MRSA treated with MZGB showed weakened green fluorescence of SYTO-9, while the red fluorescence of PI increased. SEM analysis was used to investigate the morphological changes in *S. aureus* and *E. coli*. Both *S. aureus* and *E. coli* treated with BC displayed smooth and intact structures. In contrast, *S. aureus* and *E. coli* on MZGB exhibited severe shrinkage, deformation, and rupture (Fig. 3c). Similarly, in the crystal violet staining experiment, the disruption of bacterial biofilms by MZGB increased with the concentration of MZG (Fig. 3d,g, h).

To further elucidate the antibacterial mechanism of MZG, particularly its potential independence from ROS and its behavior in acidic environments, we employed ESR spectroscopy to investigate the •OH scavenging capabilities of both MZG and glucose + MZG systems (Fig. S7c). The results demonstrated that the glucose + MZG system exhibited significantly enhanced •OH scavenging ability compared to MZG alone, indicating that the presence of glucose under acidic conditions may augment the antioxidant capacity of MZG rather than directly induce hydroxyl radical generation. These findings indicate that the antibacterial mechanism of MZG is unlikely to rely on hydroxyl radicals produced via the Fenton-like reaction. To further assess the antibacterial performance of the material, we treated bacterial suspensions with MZG, glucose + MZG, and pure ZIF-8, respectively, and quantified their antibacterial efficacy (Fig. S11). Notably, no significant differences were observed among the MZG, glucose + MZG, and pure ZIF-8 groups, indicating that the antibacterial activity of MZG primarily arises from the intrinsic antibacterial properties of ZIF-8 rather than ROS generation through Fenton-like reactions.

Compared to traditional antimicrobial strategies that rely on external stimuli such as photothermal therapy (PTT) and photodynamic therapy (PDT) [34,35], the MZGB hydrogel offers an antibacterial mechanism that operates without the need for external activation. Conventional PTT and PDT approaches typically depend on laser irradiation to induce localized heating or generate ROS to eliminate pathogens. However, these strategies face significant limitations in clinical application, including the dependence on specialized laser equipment, limited tissue penetration, and strict requirements for light exposure. These factors considerably restrict their use in complex infection environments or deep-seated tissue settings. In contrast, the MZGB hydrogel demonstrates excellent antibacterial performance under stimulation-free conditions by integrating sustained Zn^2+^ release with its intrinsic physical structure. The Zn^2+^ ions released from ZIFs adhere to bacterial membranes through electrostatic interactions, disrupting membrane integrity and causing leakage of intracellular contents, ultimately leading to bacterial death [36,37]. In addition, the nanoscale structure and surface roughness of MZG contribute to physical damage to the bacterial cell wall and membrane, further enhancing the antibacterial effect through mechanical disruption [38,39]. Furthermore, the porous network of the hydrogel generates interfacial stress during contact with bacteria, which applies continuous mechanical pressure through a "contact, adhesion, and rupture" mechanism [40]. This enables broad-spectrum, non-specific physical disruption of bacterial cells. Taken together, the MZGB hydrogel establishes an antibacterial strategy that is independent of ROS generation and external triggers. This approach not only improves antimicrobial efficacy but also significantly expands its applicability in treating deep tissue infections and complex wound environments.

### 3.5 Biocompatibility of MZGB

Previous studies have raised safety concerns regarding the absorption of nanoparticles [41]. On one hand, the cytotoxicity of nanoparticles is considered concentration-dependent. On the other hand, accumulated cytotoxic effects can vary significantly depending on the cell lines used in testing [42]. Therefore, conducting biocompatibility testing of MZG and MZGB is crucial to assess its safety profile.

We systematically evaluated the concentration-dependent cytotoxicity of MZG and MZGB. As shown in Fig. S12a–c, the CCK-8 assay results on days 1, 3, and 5 demonstrated that when the concentration of MZG nanozymes increased from 0 to 80 μg/mL, there was no significant toxicity compared with the control group. On the contrary, a remarkable increase in cell viability was observed, indicating a pronounced proliferation-enhancing effect. However, at a concentration of 160 μg/mL, cell viability decreased, suggesting that excessively high concentrations may exert an inhibitory effect on cell growth. Furthermore, the CCK-8 results of MZGB (Fig. S12d–f) demonstrated excellent biocompatibility, showing minimal impact on the viability of HUVECs, L929, and RAW264.7 cells. However, slight toxicity of MZGB3 was observed, possibly due to the high concentration of MZG. These findings clearly indicate that both MZG and MZGB possess high biosafety and favorable biocompatibility within a specific concentration range. Notably, in HUVECs and L929 cells, they exhibit a significant proliferation-promoting effect. Importantly, by precisely controlling the concentration of MZG, it is possible to maintain excellent biocompatibility while balancing the enhancement of antibacterial activity with potential cytotoxicity. Although increasing the MZG concentration contributes to improved antibacterial efficacy, it also poses a risk of elevated cytotoxicity. To achieve an optimal balance between antibacterial performance and cellular safety, we selected MZGB1 and MZGB2 for subsequent experiments.

### 3.6 MZGB protects cells and mitochondria from oxidative damage

**Fig. 4:**
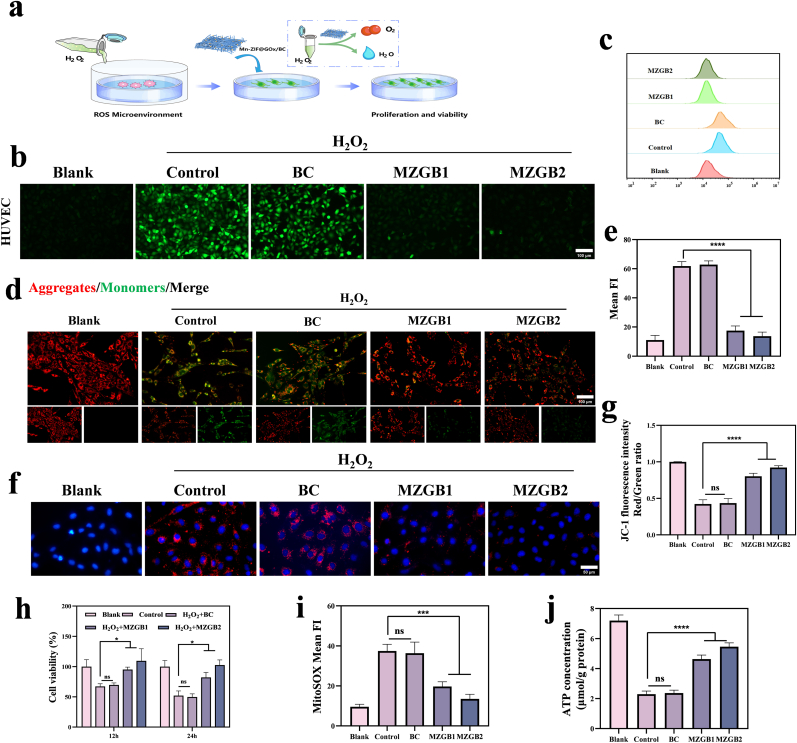
(a) Schematic illustration of the effect of MZGB on HUVECs under H_2_O_2_ stimulation. (b, e) Representative ROS staining and fluorescence intensity. (c) Flow cytometry analysis of ROS levels in HUVECs. (d,g) JC-1 assay images showing mitochondrial membrane potential and fluorescence intensity. (f, i) mtROS and fluorescence intensity. (h) Cell viability of HUVECs. (j) Expression levels of ATP in HUVECs.

After demonstrating the efficient ROS scavenging capability of MZGB, we further investigated its potential to protect cells in high ROS microenvironments (Fig. 4a). Under high glucose conditions, endothelial cells exhibit increased ROS production, leading to oxidative stress, endothelial dysfunction, and cell apoptosis [43]. Therefore, ROS clearance can alleviate oxidative stress and cell damage, promoting diabetic wound healing. To assess cellular protection, we first examined the biocompatibility of MZGB with cells in a simulated ROS environment. HUVECs exposed to high concentrations of exogenous H_2_O_2_ exhibited oxidative stress-induced cell death. In contrast, MZGB completely protected cells from H_2_O_2_-induced oxidative challenges (Fig. 4h). We then used the DCFH-DA probe to evaluate MZGB's efficiency in clearing intracellular ROS. HUVECs treated with H_2_O_2_ showed significantly increased levels of intracellular ROS, indicated by intense green fluorescence. In the BC group, this fluorescence signal did not diminish. However, following treatment with MZGB, the green fluorescence signal markedly decreased, suggesting effective mitigation of oxidative stress and cellular damage (Fig. 4b and e). Flow cytometry analysis yielded similar results (Fig. 4c). Additionally, we evaluated the protective capacity of MZGB on L929 cells, which showed consistent outcomes (Fig. S13). Similarly, the DCFH-DA probe was utilized to assess the antioxidative performance of MZGB on macrophages. Upon the addition of H_2_O_2_, the fluorescence intensity associated with ROS levels increased in the control group, indicating elevated ROS content (Fig. S14a and b). However, with MZGB treatment, this stimulus was countered, resulting in reduced oxidative stress, as evidenced by the decreased fluorescence signal. Flow cytometry analysis of fluorescence intensity also demonstrated consistent results (Fig. S14c). Thus, MZGB exhibits remarkable antioxidative properties.

Mitochondria serve as the primary source of cellular energy through ATP production, and their dysfunction not only impairs cellular metabolism but also exacerbates oxidative stress, leading to chronic inflammation and impaired tissue repair. Enhancing mitochondrial function and regulating energy metabolism are critical strategies to address delayed wound healing in diabetic patients caused by mitochondrial damage and excessive ROS accumulation [44,45].

To evaluate MMP, JC-1 dye was utilized as a detection tool. In the control group, MMP levels were high, emitting red fluorescence, whereas the H_2_O_2_-treated group exhibited lower MMP levels, indicated by green fluorescence. After treating HUVECs with MZGB, a significant increase in JC-1 aggregates and a decrease in monomers were observed, accompanied by a reduction in green fluorescence (Fig. 4d and g). Mitochondrial ROS production was subsequently assessed using Mito-Sox Red staining (Fig. 4f). The fluorescence intensity of Mito-Sox Red in the MZGB group was notably lower than that in the H_2_O_2_-treated group (Fig. 4i).

ATP, as a central molecule in cellular energy metabolism, plays a vital role in wound healing. Measurement of intracellular ATP levels revealed that oxidative stress induced by H_2_O_2_ significantly reduced ATP levels, whereas treatment with MZGB effectively restored ATP levels (Fig. 4j). This recovery of ATP production further supports the enhancement of mitochondrial function by MZGB, contributing to improved cellular viability and wound repair capacity.

MZGB effectively regulates redox balance within cells and mitochondria, eliminating excessive ROS and reducing oxidative stress-induced damage to cells. This process promotes cell proliferation, thereby facilitating repair and regeneration. Furthermore, MZGB not only demonstrates potent mtROS-scavenging capabilities but also enhances mitochondrial function and increases ATP production. By maintaining mitochondrial integrity and promoting energy homeostasis, MZGB creates a favorable environment for tissue regeneration, accelerating diabetic wound healing and improving overall outcomes.

### 3.7 Immunomodulatory abilities of MZGB

**Fig. 5:**
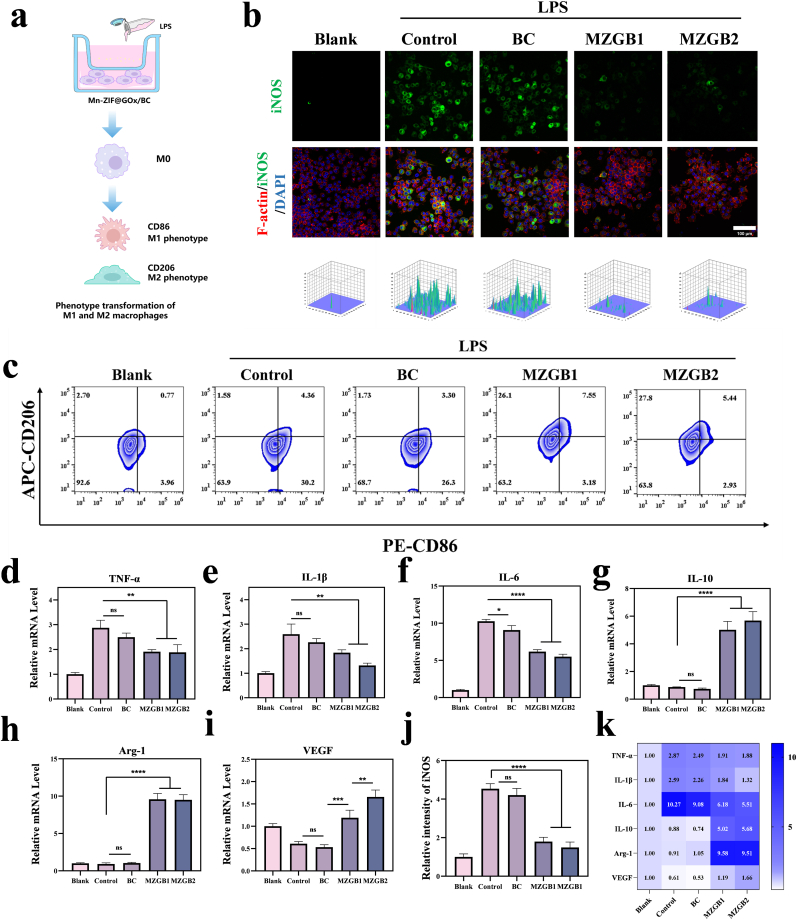
Immunomodulatory effects of MZGB. (a) Schematic illustration of LPS induction and MZGB treatment of RAW 264.7 cells. (b, j) iNOS expression in RAW 264.7 cells and statistical analysis of fluorescence intensity. (c) The expression levels of M1 (CD86+) and M2 (CD206+) macrophages across different groups were assessed using flow cytometry. (d) TNF-*α*, (e) IL-1*β*, (f) IL-6, (g) IL-10, (h) Arg-1, and (i) VEGF mRNA expression levels in RAW 264.7 cells induced by LPS and treated with different hydrogels. (k) Heatmap of mRNA expression levels in RAW 264.7 cells.

Macrophages are pivotal in regulating inflammation and facilitating wound healing [46]. The pathological conditions of diabetic wounds can exacerbate M1 macrophage infiltration, increasing inflammation and hindering the establishment of a reparative and anti-inflammatory environment necessary for effective wound healing [47]. Therefore, encouraging macrophages to polarize towards the M2 phenotype is a promising approach to accelerate the healing of chronic wounds [48,49]. We investigated the immune modulation ability of MZGB on macrophages (Fig. 5a). Flow cytometry was first employed to analyze cell phenotypes following MZGB treatment, characterized by specific biomarkers for macrophages (F4/80+), M1 phenotype (CD86+), and M2 phenotype (CD206+). As depicted in Fig. 5c, the proportion of CD86+ cells markedly increased following the addition of LPS. Following intervention with MZGB hydrogel, the proportion of CD86+ cells markedly decreased, while the proportion of CD206+ cells increased. Compared to the control group, the proportion of M1 macrophages marked by CD86+ decreased from 27.5 % to 8.26 % and 6.94 % in the MZGB1 and MZGB2 groups, respectively (Fig. S15a). Meanwhile, the proportion of M2 macrophages marked by CD206+ increased from 3.61 % to 29.3 % and 29.4 % in the MZGB1 and MZGB2 groups, respectively (Fig. S15b).

In the MZGB group, the expression levels of inflammatory factors in M1 macrophages, including TNF-*α* (Fig. 5d), IL-1*β* (Fig. 5e), and IL-6 (Fig. 5f), were significantly downregulated. In contrast, markers of M2 phenotype macrophages, including the anti-inflammatory cytokine IL-10 (Fig. 5g) and Arg-1 (Fig. 5h), were significantly upregulated following MZGB treatment. Immunofluorescence (IF) staining further indicated that MZGB significantly lowered the expression levels of iNOS (Fig. 5b and j). These results imply that MZGB effectively promotes macrophage polarization towards the M2 phenotype, enhancing its immunomodulatory capabilities and benefiting the regulation of the immune microenvironment.

Compared with conventional functional hydrogels or nanomaterials that primarily rely on a single bioactive molecule to mediate anti-inflammatory or antioxidative effects, which often results in limited therapeutic dimensions, poor sustainability, and inadequate adaptability to complex inflammatory microenvironments [50,51], the MZGB hydrogel exhibits superior immunomodulatory capabilities characterized by more systematic and coordinated mechanisms. This material simulates the enzymatic activities of SOD and CAT, enabling sustained and efficient scavenging of excessive ROS, thereby significantly alleviating oxidative stress-induced damage to immune cells and laying the foundation for microenvironmental stabilization. In addition, MZGB promotes macrophage polarization toward the M2 phenotype, which enhances the secretion of anti-inflammatory cytokines and pro-regenerative factors. This further suppresses inflammatory responses and facilitates tissue repair. Notably, these regulatory effects form a positive feedback loop. The anti-inflammatory environment established by M2 macrophages further reduces ROS production, thereby reinforcing immune homeostasis. Overall, MZGB combines ROS scavenging, immune cell reprogramming, and microenvironmental optimization into a comprehensive therapeutic strategy. It achieves self-sustained and adaptive immunomodulation, offering promising potential for treating diabetic wounds.

### 3.8 RNA sequencing (RNA-Seq) analysis of therapeutic performance of MZGB hydrogels

**Fig. 6:**
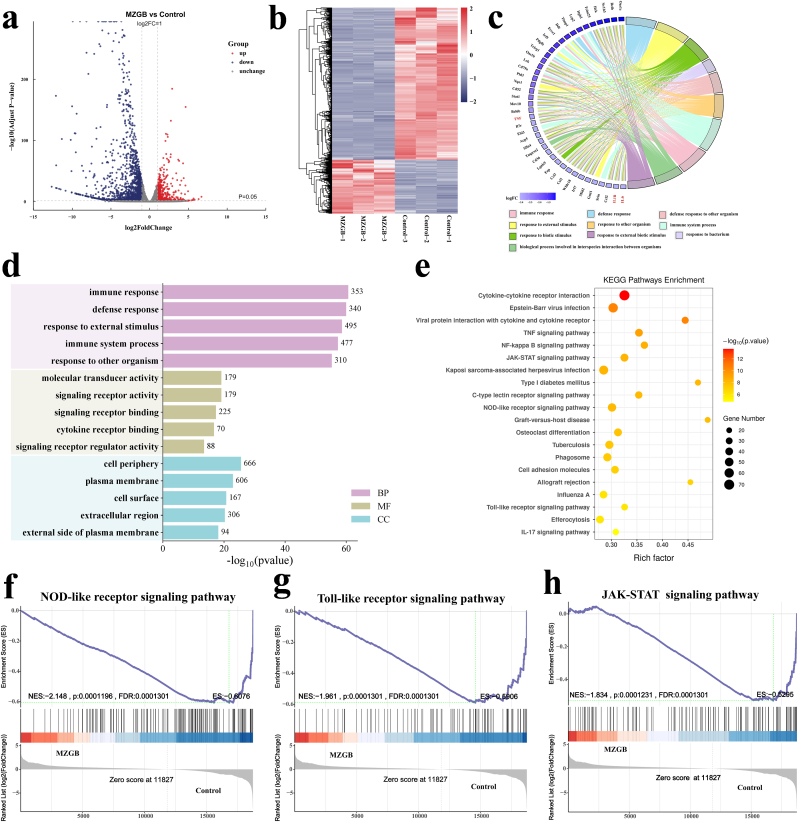
Transcriptome sequencing analysis of RAW 264.7 cells treated with MZGB hydrogel. (a) Volcano plot showing DEGs. (b) Heatmap of DEGs. (c) Chord diagram showing GO enriched terms and their corresponding downregulated genes. (d) GO enrichment analysis showing relevant pathways. (e) KEGG pathway enrichment analysis of DEGs. (f) GSEA of the NOD-like receptor signaling pathway. (g) GSEA of the Toll-like receptor signaling pathway. (h) GSEA of the JAK-STAT signaling pathway.

To assess the mRNA expression profile of macrophages exposed to LPS-induced inflammatory conditions, we employed RNA-Seq (Fig. 6) on cells cultured on the MZGB hydrogel. Compared to the control group, the MZGB-treated group demonstrated significant differential gene expression, with a total of 2236 genes identified as differentially expressed genes (DEGs), comprising 584 upregulated and 1652 downregulated genes (Fig. 6a). Fig. 6b presents a heatmap depicting the expression patterns of the 2236 DEGs, clearly highlighting the pronounced differences in gene expression between the control and MZGB groups. Gene Ontology (GO) analysis indicated that the biological processes were predominantly related to immune responses, defense mechanisms, and immune system activities (Fig. 6d). Notably, downregulated genes such as IL-6, IL-1*β*, and TNF-*α* were observed (Fig. 6c). To clarify the cellular signaling pathways modulated by the DEGs, we conducted Kyoto Encyclopedia of Genes and Genomes (KEGG) pathway enrichment analysis. Key pathways related to immune regulation, including the TNF signaling pathway, NF-*κ*B signaling pathway, and JAK-STAT signaling pathway, were prominently identified (Fig. 6e).

To further investigate the pathways activated by MZGB, we utilized gene set enrichment analysis (GSEA) to examine the relevant signaling pathways. The analysis revealed that in MZGB-treated cells, the NOD-like receptor signaling pathway (Fig. 6f), Toll-like receptor signaling pathway (Fig. 6g), and JAK-STAT signaling pathway (Fig. 6h) were notably downregulated. These biological processes, which play crucial roles in inflammation, were significantly inhibited. MZGB reduces ROS production, thereby diminishing the activation of NOD-like receptor and Toll-like receptor-mediated inflammatory responses, ultimately leading to the suppression of excessive pro-inflammatory cytokine release. Notably, MZGB negatively regulates signal transducers and activators of transcription Stat1 and Stat2, as well as interferon regulatory factor Irf9 (Fig. S16). According to previous reports, Stat1, Stat2, and Irf9 together form the interferon-stimulated gene factor 3 (ISGF3) complex [52,53]. MZGB's negative regulation of the ISGF3 complex likely inhibits interferon signaling pathways and reduces the expression of pro-inflammatory genes, thereby modulating immune responses and inflammatory reactions. Furthermore, MZGB is also capable of negatively regulating the JAK-STAT signaling pathway, which may further help reduce cytokine storms and associated tissue damage caused by excessive inflammation. The combined action of these mechanisms contributes to MZGB's ability to modulate immune responses and alleviate inflammation.

### 3.9 Angiogenesis and cell migration after antioxidant treatment *in vitro*

Previous research has demonstrated that macrophages that enhance wound healing can stimulate angiogenesis, thus bridging the transition from initial inflammation to later stages of tissue regeneration and repair [54,55]. After confirming the excellent ROS scavenging capability of MZGB and its improvement of HUVECs viability under high ROS conditions, we further evaluated the regenerative potential of MZGB *in vitro*, focusing on the ability of macrophage modulation to promote tissue regeneration.

**Fig. 7:**
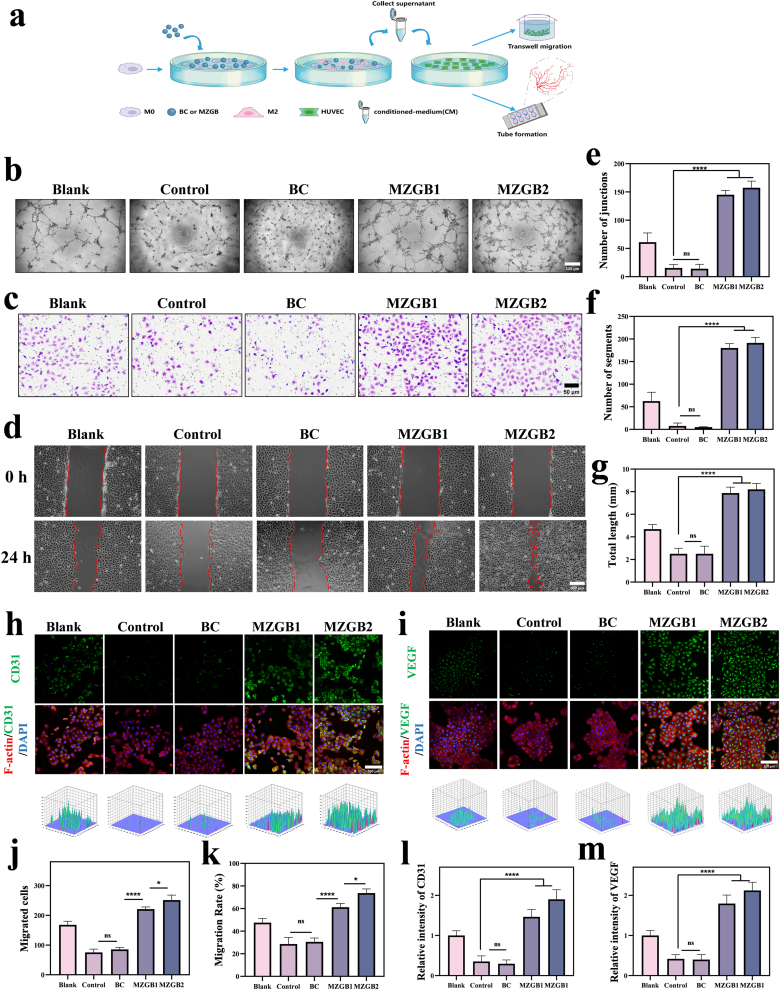
Tube formation, migration, scratch assay, and IF images of HUVECs under different CM. (a) Schematic illustration of obtaining CM for evaluating HUVECs' migration and tube formation. (b) Tube formation of HUVECs in CMs. (e, f, g) Number of junctions, segments, and total length with statistical analysis. (c, j) Transwell migration results of HUVECs in CMs. (d, k) Scratch assay results of HUVECs in CMs. (h, l) CD31 expression and fluorescence intensity in HUVECs in CMs. (i, m) VEGF expression and fluorescence intensity in HUVECs in CMs.

Angiogenesis and the migration of endothelial cells and fibroblasts were assessed using a macrophage CM (Fig. 7a). First, we conducted a tube formation assay to simulate angiogenesis *in vitro*. Unsurprisingly, we found that the control and BC groups had significantly impaired blood vessel formation, with almost no effective vascular structures, mostly forming scattered nodules or dendritic projections (Fig. 7b). However, in the MZGB1 and MZGB2 groups, lumen formation was clearly observed, accompanied by an increase in total tube length, junction points, and the number of segments (Fig. 7b,e, f, g). The MZGB groups demonstrated the best pro-angiogenic effects in terms of angiogenesis capability. Additionally, IF staining for VEGF and CD31 expression in HUVECs showed more significant signals in the MZGB groups (Fig. 7h, i, l, m). During wound healing, endothelial cell migration is a crucial step in angiogenesis. The impact of MZGB on cell migration was evaluated through Transwell and scratch assays. In the control group, HUVECs migration was hindered (Fig. 7c and j). The MZGB groups exhibited stronger migratory activity compared to the other groups. Simultaneously, scratch assays using HUVECs demonstrated that MZGB had the most significant impact on scratch closure (Fig. 7d and k).

Fibroblasts are essential in the wound-healing process, promoting tissue repair, wound contraction, and the remodeling of healed tissue. We found that the migration of L929 cells was hindered in a high ROS environment. In contrast, MZGB processing significantly enhanced the migration of L929 cells (Fig. S17a and c). This finding is consistent with the results observed in HUVECs. Additionally, IF staining revealed that VEGF expression was more prominent in the MZGB group (Fig. S17b and d).

To determine whether this phenomenon is attributable to MZGB's stimulation of RAW264.7 cells to secrete factors that regulate cell behavior, we assessed VEGF levels in RAW264.7 cells after MZGB treatment. qRT-PCR results indicated that MZGB treatment promoted VEGF secretion by RAW264.7 cells (Fig. 5i), thereby enhancing the migration and tube formation abilities of HUVECs. As a result, the CM treated with MZGB hydrogel markedly stimulated angiogenesis *in vitro*, indicating that MZGB hydrogel enhances the angiogenic potential of HUVECs through paracrine factors secreted by macrophages. This finding underscores the considerable potential of MZGB hydrogel in advancing wound healing.

These results indicate that MZGB improves the functionality of fibroblasts and endothelial cells by mitigating oxidative stress and facilitating the polarization of macrophages to the M2 phenotype. This modulation fosters a tissue microenvironment that supports cell migration, differentiation, and regeneration, thereby underscoring the potential of MZGB to enhance tissue regeneration. Additionally, newly formed blood vessels enable the transport of additional macrophages to the injured area. Consequently, MZGB enhances the interplay between vascular endothelial cells, macrophages, and fibroblasts, resulting in a positive feedback loop that promotes diabetic wound healing.

### 3.10 Evaluation of diabetic wound healing ability of MZGB *in vivo*

To further evaluate the wound healing efficacy of MZGB hydrogel, we created a full-thickness skin wound model in diabetic rats, employing a 10 mm diameter wound on the dorsal region of the rats. The rats were randomly assigned to one of four groups: Normal, diabetes mellitus (DM), BC, and MZGB. The Normal group comprised healthy rats, while the DM, BC, and MZGB groups included diabetic rats. All animal procedures were approved by the Animal Ethics Committee of Shanxi Medical University (Ethics Approval No.: KQDW-2024-004).

**Fig. 8:**
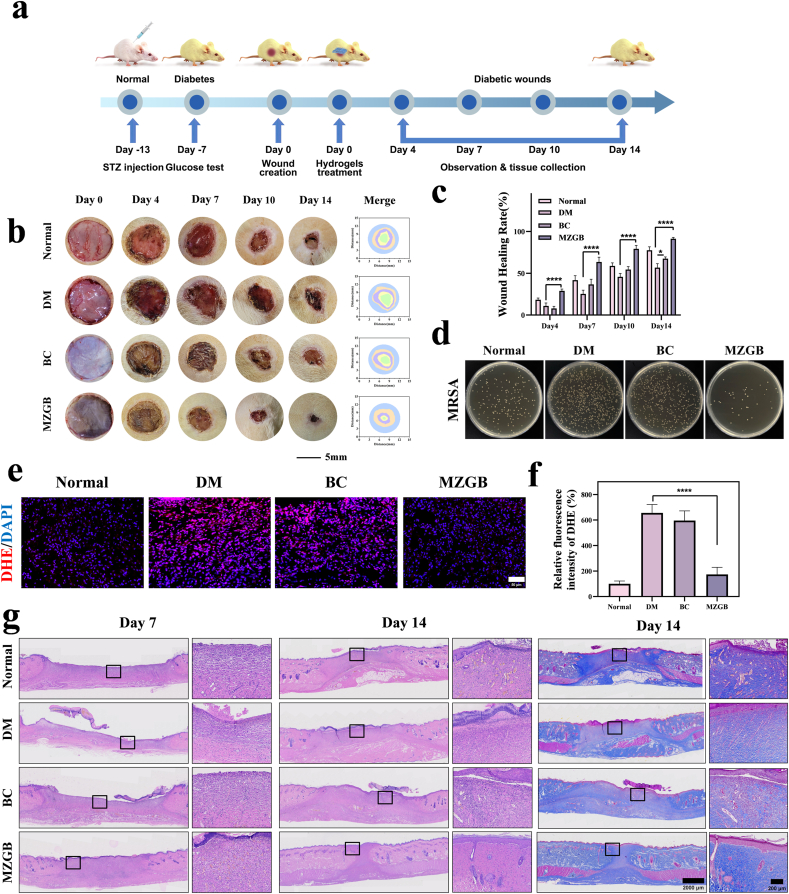
Effect of MZGB on wound healing in diabetic rats. (a) The diabetic rat model establishment, wound formation. (b) Representative images showing wound healing across different groups, along with a schematic representation of the wound healing process. (c) Wound healing rates at various time points for each group. (d) Representative images of bacterial colonies from the wound site on agar plates after different treatments. (e, f) DHE fluorescence images with corresponding fluorescence intensity quantification. (g) Representative H&E and Masson's trichrome staining images of wound samples from different groups on days 7 and 14.

Fig. 8a depicts the progression of full-thickness skin wound healing at days 0, 4, 7, 10, and 14. Although the overall trend in wound healing was similar across the four groups, the rate of healing varied significantly (Fig. 8b). Wound healing rates were quantified, as illustrated in Fig. 8c. On day 7, the wound healing rate in the MZGB hydrogel-treated group reached 63.48 %, significantly surpassing that of the other groups. In contrast, the DM group had a healing rate of only 25.29 %, significantly lower than the 41.7 % observed in the normal group. Additionally, purulent exudate was noted in the wounds of the DM group. After 10 days of treatment, the wound area in all groups was further reduced. The MZGB group demonstrated a significantly higher healing rate, while the DM group still had a substantial unhealed wound area. Compared to the DM group, the BC group exhibited a slightly improved healing rate. This enhancement is likely due to the excellent water absorption capacity of BC, which effectively absorbs wound exudate and maintains a moist wound environment, thereby promoting cell migration and tissue regeneration. Furthermore, wound tissue treated with MZGB hydrogel exhibited high hair coverage, and the newly formed skin closely resembled the surrounding normal tissue, indicating a superior wound healing outcome.

To further evaluate the *in vivo* antibacterial efficacy of the MZGB hydrogel, an MRSA bacterial suspension (10 μL, 1 × 10^8^ CFU/mL) was directly applied to the wound area. MRSA colony counting was performed on wound tissues on day 7 post-treatment (Fig. 8d and S18). The results revealed a substantial bacterial burden in the DM group, with a high density of MRSA colonization. In contrast, MZGB treatment significantly reduced MRSA colonization, demonstrating superior antibacterial activity. The BC group exhibited a lower MRSA colony count than the DM group, likely due to its function as a wound dressing, which partially prevents external bacterial invasion and limits MRSA spread. However, compared to MZGB, the antibacterial effect of BC remained limited.

Skin section analysis was performed to assess the wound reconstruction, antioxidant, anti-inflammatory, and pro-angiogenic abilities of the MZGB hydrogel. First, the structural reconstruction of the regenerated skin tissue was analyzed using H&E and Masson staining (Fig. 8g). H&E staining revealed significant inflammatory regions and an incomplete epithelial layer in the DM group on day 7. In contrast, the wound tissue treated with MZGB exhibited a thick layer of regenerated granulation tissue. By day 14, the MZGB group displayed complete and continuous epithelial tissue, with the most notable re-epithelialization. Additionally, newly formed sebaceous glands and hair follicles were observed, resembling normal skin tissue. Masson staining demonstrated that wounds treated with MZGB had the highest collagen content, with significantly increased collagen fiber density and prominent regeneration of skin appendages. The collagen fibers in the MZGB group displayed a more regular topology and alignment of collagen networks. In contrast, the fiber arrangement in the other groups was relatively loose and underdeveloped.

To evaluate the ROS scavenging capacity *in vivo*, we employed a dihydroethidium (DHE) probe to measure ROS levels within the wounds (Fig. 8e). The DM group exhibited bright red fluorescence signals, indicating high ROS levels. In contrast, the MZGB group showed significantly reduced red fluorescence. Quantitative analysis further confirmed that ROS levels were lower in the MZGB group compared to the DM group (Fig. 8f), suggesting that MZGB effectively scavenges excess ROS and alleviates oxidative stress.

### 3.11 Immunofluorescence analysis

**Fig. 9:**
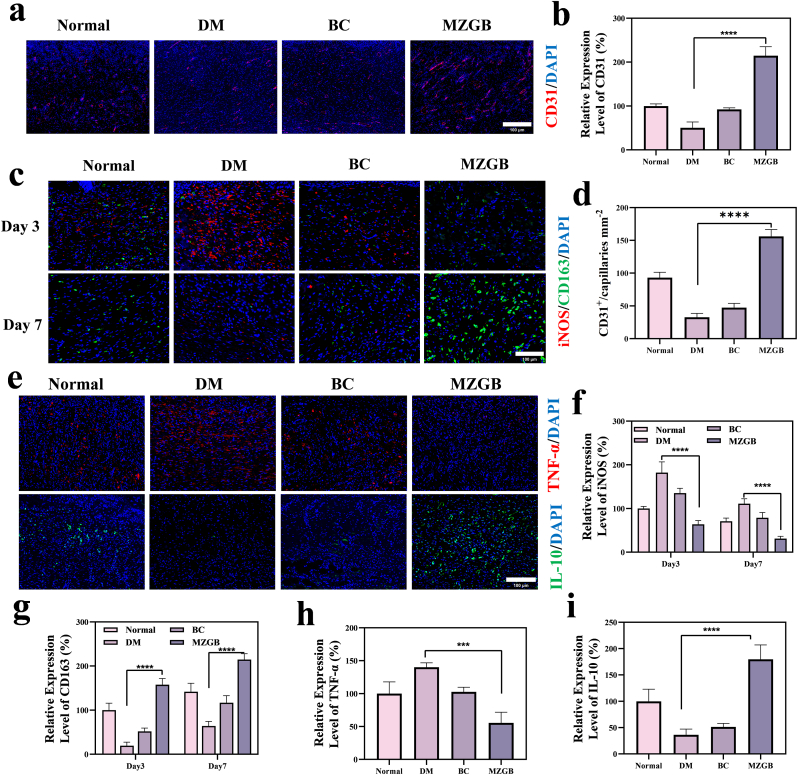
IF staining of wound tissue. (a, b, d) Representative CD31 IF images and corresponding quantitative analysis. (c, f, g) Representative iNOS/CD163 IF images and corresponding quantitative analysis in wound tissue on days 3 and 7. (e, h, i) Representative TNF-*α* and IL-10 IF images and corresponding quantitative analysis in wound tissue on day 7.

In a normal wound microenvironment, macrophages can polarize to the anti-inflammatory M2 type, thereby promoting tissue healing. However, due to the accumulation of glycosylation residues and hyperglycemia, macrophages in diabetic wounds fail to polarize to the M2 type. The increased presence of M1 macrophages prolongs inflammation in diabetic wounds [56,57]. To further elucidate the impact of MZGB on macrophage polarization, we performed IF staining to examine macrophage infiltration within the wounds. As illustrated in Fig. 9c, the DM and BC groups displayed significantly elevated levels of iNOS-positive M1 macrophages compared to the other groups. Conversely, the MZGB group had significantly fewer iNOS-expressing M1 macrophages (Fig. 9f). Additionally, the MZGB group showed a higher presence of CD163-positive M2 macrophages (Fig. 9g).

Inflammatory factors secreted by M1 macrophages, such as TNF-*α*, are associated with prolonged excessive inflammatory responses that impede the healing process. In contrast, M2 macrophages secrete substantial amounts of anti-inflammatory cytokines and tissue repair factors, including IL-10. We analyzed the levels of inflammation-related cytokines through IF staining. As shown in Fig. 9e, the DM group exhibited the highest expression levels of TNF-*α* and the lowest expression levels of IL-10. Additionally, compared to the normal, DM, and BC groups, the MZGB group displayed reduced expression of TNF-*α* (Fig. 9h) and increased expression of IL-10 (Fig. 9i). These results suggest that MZGB effectively facilitates the polarization of macrophages towards the M2 phenotype, thereby achieving a favorable therapeutic effect on diabetic inflammatory wounds.

Persistent inflammation leading to impaired angiogenesis is a crucial factor contributing to the non-healing of diabetic wounds. Therefore, we examined the extent of neovascularization in newly formed tissues across different groups. CD31, a specific marker for vascular endothelium, was used to assess the level of angiogenesis through IF staining. Notably, MZGB significantly promoted the expression of CD31, with significantly higher microvascular density observed in the MZGB group (Fig. 9a, b, d). This finding indicates that MZGB is highly effective in initiating the growth of new blood vessels. Consequently, the MZGB hydrogel accelerated angiogenesis in diabetic wounds, further enhancing the healing process *in vivo*.

Additionally, the biosafety of MZGB was evaluated by performing H&E staining on major organs, including the heart, liver, spleen, lungs, and kidneys (Fig. S19). No significant histological abnormalities were observed in these tissues, indicating that MZGB exhibits excellent biocompatibility.

### *3.12* *Clinical translation prospects of* MZGB *hydrogel*

The clinical translation of bioengineered hydrogels for diabetic wound healing requires careful consideration of scalability, regulatory approval, and cost-effectiveness. While MZGB hydrogel demonstrates promising therapeutic effects in laboratory and animal models, ensuring its feasibility for real-world applications is crucial.

One critical aspect of clinical translation is the scalability of MZGB hydrogel production. The synthesis process is straightforward, reproducible, and primarily involves self-assembly and electrostatic interactions. The key components, including BC and Mn-ZIF, are derived from scalable sources. BC can be mass-produced through microbial fermentation, and MOF-based nanozymes have demonstrated large-scale synthesis in other studies. Optimizing reaction parameters, such as temperature, reaction time, and precursor concentrations, could further facilitate industrial-scale manufacturing.

From a regulatory perspective, MZGB hydrogel components have shown biocompatibility in previous studies. Both BC and Zn-based MOFs have shown significant biomedical potential. Nevertheless, additional preclinical toxicity assessments, degradation studies, and long-term stability evaluations are required to meet clinical translation standards. Future studies will focus on meeting Good Manufacturing Practice standards to ensure regulatory compliance.

The fabrication of MZGB hydrogel is relatively cost-effective, as its major raw materials are commercially available and scalable. Further optimization of synthesis parameters and automation could reduce production costs, enhancing its feasibility for clinical applications. Additionally, the hydrogel's multifunctional benefits, including ROS scavenging, antibacterial properties, and promotion of angiogenesis, justify its production costs when compared to conventional wound dressings.

In summary, while MZGB hydrogel shows significant promise for diabetic wound treatment, further investigations into scalability, regulatory compliance, and cost-effectiveness are necessary to facilitate its clinical translation. Addressing these challenges will pave the way for the hydrogel's successful integration into real-world medical applications, ultimately improving patient outcomes.

## 4 Conclusion

In this study, we designed an MZGB hydrogel to promote diabetic wound healing. This hydrogel rapidly consumes glucose within the wound microenvironment while exhibiting SOD- and CAT-like activities, demonstrating superior ROS-scavenging capabilities. Notably, it protects mitochondrial function and regulates energy metabolism. MZGB induces M2 macrophage polarization, enhances intercellular communication, and stimulates a regenerative environment enriched with pro-angiogenic factors, thereby promoting the proliferation and migration of fibroblasts and endothelial cells, as well as facilitating *in vitro* angiogenesis and normal tissue repair. Additionally, we observed that the MZGB hydrogel exhibits robust ROS-independent antibacterial properties. *In vivo* experiments demonstrated that MZGB hydrogel effectively scavenges ROS, restores immune and redox homeostasis, and modulates the inflammatory microenvironment through macrophage reprogramming, ultimately promoting angiogenesis and accelerating diabetic wound healing. Therefore, the MZGB hydrogel demonstrates remarkable efficacy in enhancing diabetic wound repair and holds significant potential for immune-regulation-based wound therapies.

## CRediT authorship contribution statement

**Jingyu Yan:** Writing – original draft, Conceptualization. **Yifan Zhao:** Methodology, Investigation. **Chenying Cui:** Methodology, Investigation. **Lihong Zhou:** Methodology, Investigation. **Yurong Xu:** Resources, Investigation. **Ziyang Bai:** Resources, Investigation. **Kaifang Zhang:** Resources, Investigation. **Jiahui Tong:** Formal analysis. **Yingyu Liu:** Formal analysis. **Lingxiang Sun:** Formal analysis. **Meijun Du:** Data curation. **Yanling Mi:** Data curation. **Xing Wang:** Writing – review & editing. **Xiuping Wu:** Project administration, Funding acquisition. **Bing Li:** Project administration, Funding acquisition.

## Declaration of competing interest

The authors declare that they have no known competing financial interests or personal relationships that could have appeared to influence the work reported in this paper.

## Data Availability

Data will be made available on request.
