# Supplementary material for: Dynamic multistage nanozyme hydrogel reprograms diabetic wound microenvironment: synergistic oxidative stress alleviation and mitochondrial restoration
Source: Mater Today Bio. 2025 Apr 17;32:101780. doi: 10.1016/j.mtbio.2025.101780 (PMC12032933; doi:10.1016/j.mtbio.2025.101780)
Supplement: Multimedia component 1 [file mmc1.docx]

**Supporting Information**

**Figures**

**Fig. S1.** SEM Images. (a) SEM image of Mn-ZIF. (b) SEM image of ZIF-8.

**Fig. S2.** Particle size distribution of MZG as determined by DLS.

**Fig.** **S3.** TEM and EDS elemental maps of MZG.

**Fig.** **S4.** High-resolution XPS spectra of Zn 2p (a), Mn 2p (b), and S 2p (c) of MZG.

**Fig.** **S5.** DPPH scavenging rate of MZG.

**Fig.** **S6.** Assessment of glucose consumption (a) and pH change capacity (b) of MZG in the presence of glucose.

**Fig. S7.** Evaluation of ROS clearance in MZG in the presence of glucose. (a) The SOD-like activity was detected by NADH-mPMS-NBT method. (b) MZG catalyzes the change of the amount of O_2_ generated by H_2_O_2_. (c) DMPO/·OH ESR spectrum of MZG.

**Fig.** **S8.** Water contact angle images.

**Fig.** **S9.** (a) The antibacterial performance of MZGB against MRSA was evaluated by plate colony counting and live/dead bacterial staining. (b) Statistical analysis of the survival rate from plate colony counting was performed.

**Fig.** **S10.** Quantitative analysis of inhibition zone diameters.

**Fig.** **S11.** Exploring the antibacterial mechanism of MZG. (a) The antibacterial activity of MZG, glucose + MZG, and ZIF-8 against *S.aureus* and *E.coli* was evaluated by plate counting. (b, c) Statistical analysis of the survival rate from plate colony counting was performed.

**Fig. S12.** Biocompatibility evaluation using the CCK-8 assay. (a) The viability of HUVECs cultured with different concentrations of MZG. (b) The viability of L929 cells cultured with different concentrations of MZG. (c) The viability of RAW264.7 cells cultured with different concentrations of MZG. (d) The viability of HUVECs cultured with different hydrogels. (e) The viability of L929 cells cultured with different hydrogels. (f) The viability of RAW264.7 cells cultured with different hydrogels.

**Fig.** **S13.** MZGB protects L929 cells from oxidative stress-induced damage *in vitro*. (a, b) Representative ROS staining (green fluorescence) and fluorescence intensity of untreated L929 cells or L929 cells treated with BC or MZGB under H_2_O_2_ incubation. (c) Cell viability of L929 cells under different treatment conditions.

**Fig.** **S14.** MZGB protects RAW264.7 cells from oxidative stress-induced damage *in vitro*. (a, b) Representative ROS staining (green fluorescence) and fluorescence intensity of untreated RAW264.7 cells or RAW264.7 cells treated with BC or MZGB under H_2_O_2_ incubation. (c) Flow cytometry analysis of ROS levels in RAW264.7 cells.

**Fig.** **S15.** Flow cytometry analysis of CD86+ (a) and CD206+ (b) expression in RAW264.7 cells cultured with different samples.

**Fig.** **S16.** Fold change of gene expression in the NOD-like receptor signaling pathway.

**Fig.** **S17.** Migration and IF images of L929s in CM. (a, c) Migration assay results of L929 cells in CMs. (b, d) VEGF expression and fluorescence intensity analysis of L929 cells in CMs.

**Fig.** **S18.** Quantitative analysis of antibacterial effect after treatment with different hydrogels.

**Fig.** **S19.** H&E staining of the major organs in different groups.

**Table**

**Table S1.** Primers used in the qRT-PCR analysis.

| Genes | Forward primer | Reverse primer |
| --- | --- | --- |
| TNF-*α* | CTTGTTGCCTCCTCTTTTGCTTA | CTTTATTTCTCTCAATGACCCGTAG |
| IL-1*β* | TGTGTTTTCCTCCTTGCCTCTGAT | TGCTGCCTAATGTCCCCTTGAAT |
| IL-6 | ATA ACC ACC CCT GAC CCA AC | CCC ATG CTA CAT TTG CCG AA |
| IL-10 | GGAAGACAATAACTGCACCCACT | CAACCCAAGTAACCCTTAAAGTCC |
| Arg-1 | CCCAGCTTGTCTACTTCAGTCATG | GGCAACCTGTGTCCTTTCTCCT |
| VEGF | GTCCTCTCCTTACCCCACCTCCT | CTCACACACACAGCCAAGTCTCCT |
| GAPDH | AGAAGGTGGTGAAGCAGGCATC | CGAAGGTGGAAGAGTGGGAGTTG |
